# DNA based multi-copper ions assembly using combined pyrazole and salen ligandosides†
†Electronic supplementary information (ESI) available. See DOI: 10.1039/c4sc01567c



**DOI:** 10.1039/c4sc01567c

**Published:** 2014-08-11

**Authors:** Meng Su, María Tomás-Gamasa, Thomas Carell

**Affiliations:** a Department of Chemistry , Ludwig-Maximilians-University Munich , Butenandtstraße 5-13 , 81377 , Munich , Germany . Email: thomas.carell@cup.uni-muenchen.de

## Abstract

The DNA structure is an ideal building block for the construction of functional nano-objects. In this direction, metal coordinating base pairs (ligandosides) are an appealing tool for the future specific functionalization of such nano-objects. We present here a study, in which we combine the metal ion coordinating pyrazole ligandoside with the interstrand crosslinking salen ligandoside system. We show that both ligandosides, when combined, are able to create stable multi-copper ion complexing DNA double helix structures in a cooperative fashion.

## Abstract 2

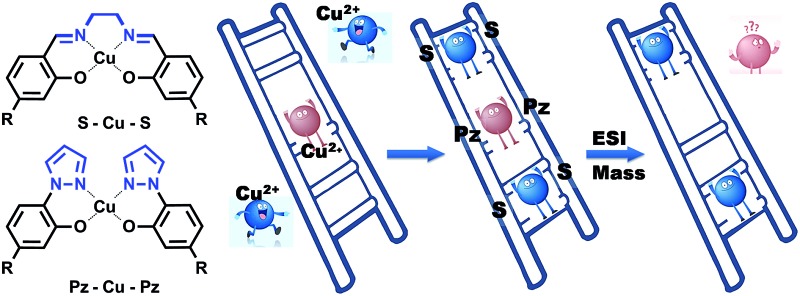
The pyrazole and salen ligandosides, when combined, are able to create stable multi-copper ion complexing DNA duplex structures in a cooperative fashion.

## Introduction

The Watson–Crick base pairing of DNA is a phenomenon that is more and more exploited for the construction of complex two and three dimensional self-assembled nano-objects.^1–3^ Nowadays, sophisticated technologies provide scientists almost total control of the self-assembly process so that basically any desired nano-object can be created using DNA as the building block.^4–6^ The next phase in the field is the functionalization of these objects, thus facilitating access to materials of relevance, *e.g.* “intelligent” drug delivery devices^7^ or building blocks useful in nano-machines.^8^ One approach for the construction of alternative and functional DNA structures was based on the design of bases showing novel base-pairing schemes,^9–11^ including those held together by hydrogen bonding patterns different from those of the natural base pairs^12^ or by simply hydrophobic interactions.^13,14^ The development of metal-mediated base pairs (ligandosides) represents a research direction of particular interest in respect to the desired functionalization of DNA.^15–17^ They can add properties to DNA, such as conductivity or magnetism,^18–20^ or they can be used to construct DNA able to perform logic operations.^21,22^

Metal base pairs comprise natural or artificial nucleobases that are able to coordinate a central metal ion. The first group involves the conversion of a natural mismatched base pair into a metal coordinated pair. Examples include the coordination of Hg^2+^ and Ag^+^ by T–T, U–U or C–A mismatches which goes in hand with the formation of stable metal base pairs.^23–26^

Regarding the artificial base pairs, a plethora of different metal modified double helixes were generated depending on the choice of the synthesized ligandoside. Diverse systems, including monodentate,^27,28^ bidentate^29–34^ as well as homo tridentate ligandosides,^35–38^ which can complex a variety of metal ions (*e.g.*, Cu^2+^, Ni^2+^, Pd^2+^) have been reported to date. Fig. S1† summarizes current reported structures. Last examples comprise new bimetal base pairs,^39,40^ which in principle would allow to construct DNA structures with heavy metal ion loading.

**Fig. 1:**
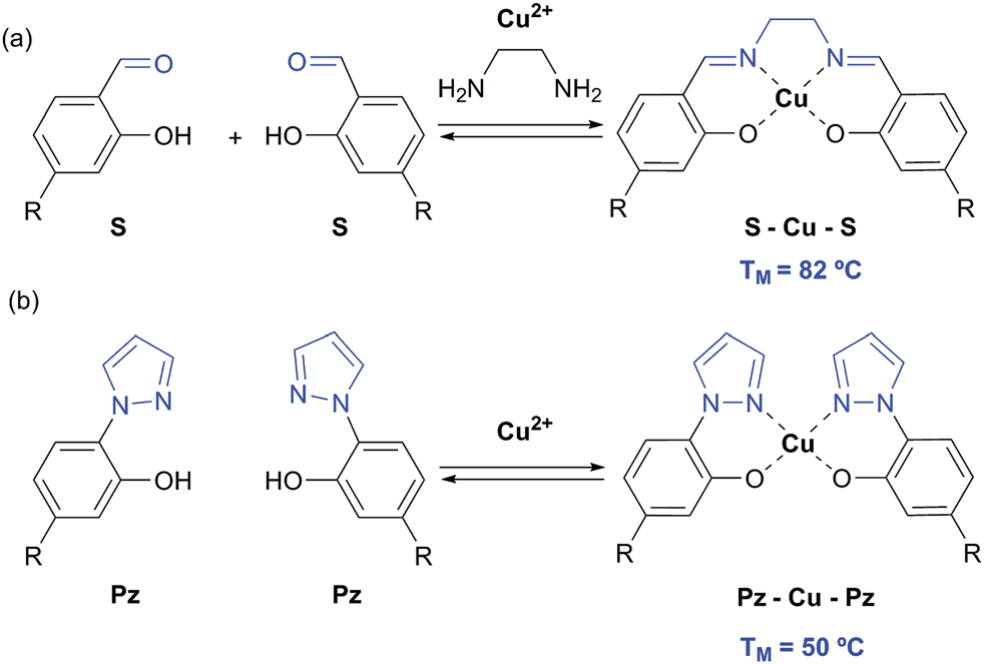
Depiction of (a) the reversible salen (S) self-base pair; (b) the pyrazole (Pz) self base pair. R = deoxyribose.

In 2005, we introduced the salen concept for the construction of a new metal base pair.^41–45^ This ligandoside involves formation of a covalent linkage between the two strands *via* a bridge established by ethylenediamine, as depicted in Fig. 1a. Due to the formed interstrand crosslink, the salen base pair was proven to be extremely stable after coordination of a copper ion.

In order to broaden this concept and to move away from the linking character of the salen system, we recently reported the pyrazole base pair (Pz) displayed in Fig. 1b.^46^ This ligandoside construction lacks the bridge but still retains the same metal coordination geometry.

Here we present stability and selectivity studies with the Pz ligandoside. We report that by combining the Pz and the salen ligandosides the generation of stable multi-Cu^2+^ complexing DNA duplexes becomes possible.

## Results and discussion

We synthesized the pyrazole ligandosides Pz and Pm according to our published procedure.^46^ Table 1 summarizes the modified DNA single strands prepared for this work.

**Table 1:** Depiction of the ligandosides Pz and Pm together with the synthesized oligonucleotides needed for this study

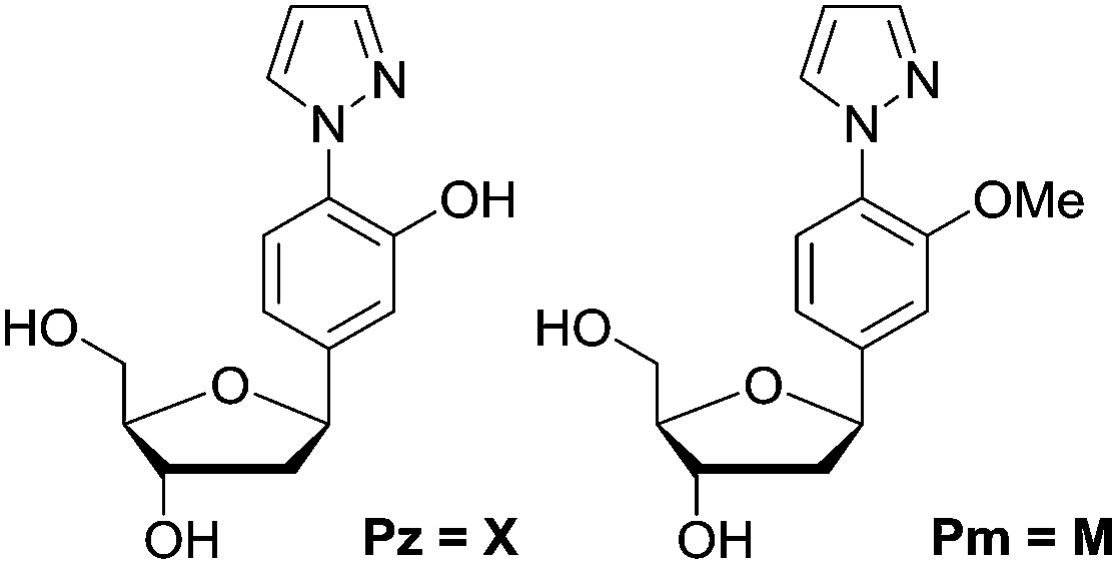			
No.	5′ ------ 3′^*a*^	Mass (calc.)	Mass^*b*^ (exp.)
0a	CAC ATT AGT GTT GTA	4579.8	4576.5
0b	CAC ATT ACT GTT GTA	4557.8	4553.7
1a	CAC ATT AXT GTT GTA	4588.8	4585.0
1b	TAC AAC AXT AAT GTG	4606.8	4602.9
2a	CAC ATT AMT GTT GTA	4602.8	4597.9
2b	TAC AAC AMT AAT GTG	4620.9	4616.3
3a	CAC ATT XXT GTT GTA	4613.8	4611.6
3b	TAC AAC AXX AAT GTG	4640.9	4636.1
4a	GCGCC XXXXXXXXXX GGCCG	6449.2	6452.4
4b	CGGCC XXXXXXXXXX CGCGC	6369.2	6370.8
5a	CAC STT AXT GTS GTA	4571.8	4570.0
5b	TAC SAC AXT AAS GTG	4589.8	4586.8
6a	CAC STT AMT GTS GTA	4585.8	4586.8
6b	TAC SAC AMT AAS GTG	4603.8	4604.2
7a	CATGSTXGSAXCSTXCSTGCA	6451.1	6450.7
7b	TGCASGXASGXTSCXASCATG	6500.1	6500.1
8a	GCGCG XXXXX GGCCG	4758.9	4757.6
8b	CGGCC XXXXX CGCGC	4678.9	4676.6

^*a*^X = Pz, M = Pm, S = salen base.

^*b*^Data from MALDI-TOF mass spectrometry.

### Complexation properties

To determine the thermal stability of DNA duplexes containing the ligands Pz and Pm, melting points were measured in the absence and in the presence of copper ions.

For duplexes **1a**/**1b** and **2a**/**2b** having one artificial base pair inserted, a decrease of the melting point value from 49 °C (*T*_M_ of the reference duplex **0a**/**0b** containing a GC base pair) to 41 °C and 42 °C was observed, respectively. Upon metal complexation, the duplex with the Pz–Cu–Pz complex furnished a melting temperature of 50 °C, slightly above the original *T*_M_ of the duplex **0a**/**0b**. In contrast, no increase of the *T*_M_ value was detected for duplex **2a**/**2b** (42 °C). This result shows that the presence of the phenolic hydroxyl groups and in particular, their ability to become deprotonated, are critical for metal complexation, which confirms the coordination geometry proposed in Fig. 1b.

**Fig. 2:**
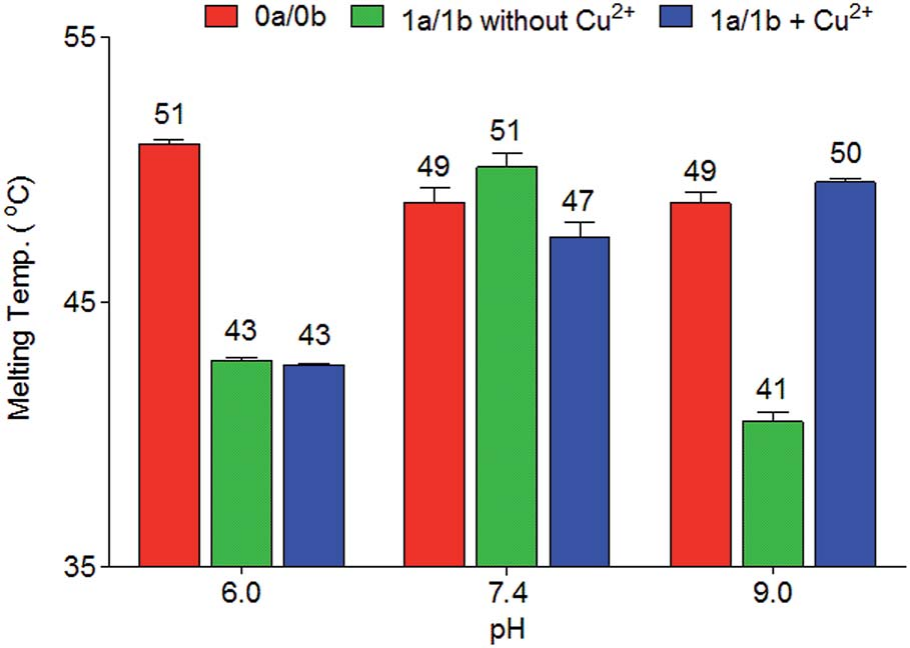
Melting temperatures of duplexes **0a**/**0b** and **1a**/**1b** at different pH values. Conditions: 150 mM NaCl, 10 mM Na_2_HPO_4_/NaH_2_PO_4_ buffer pH 6.0/7.4 or CHES buffer pH 9.0, 1 μM oligonucleotide, with or without 1 μM Cu^2+^, final volume of 200 μL.

In order to investigate in more detail the need for deprotonation, a study of the relationship between duplex thermostability and the pH value was carried out, using duplexes **1a**/**1b** and **2a**/**2b**. The data are compiled in Fig. 2, S2 and S3 and Table S1.† At pH 6.0, no substantial differences between the presence and absence of copper ions were found. The *T*_M_ is approximately 8 °C lower than the *T*_M_ of the canonical duplex **0a**/**0b**. At this pH, the phenolic hydroxyl groups are obviously not deprotonated, which blocks Cu^2+^ complexation. Interestingly, at physiological pH 7.4, a slight difference (51 °C to 47 °C) regarding the melting point values between **1a**/**1b** without and with Cu^2+^ was observed. We speculate that, at this pH, half deprotonation takes place, which leads, in the absence of Cu^2+^, to the formation of a stabilizing H-bonding bridge between the Pz–Pz base pair. Upon addition of Cu^2+^, the copper base pair is formed and is as stable as the H-bonded Pz–Pz self pair. At pH 9.0, the metal-free duplex is less stable, likely because of phenoxide formation followed by charge repulsion. Addition of Cu^2+^ stabilizes the system, in agreement with formation of the Pz–Cu–Pz base pair.

The overall duplex conformation was further investigated by circular dichroism spectroscopy. All measured CD spectra of DNA containing the Pz ligandoside in the absence and presence of Cu^2+^ support the presence of a B-form helix (Fig. S4 and S5†).

### Selectivity of the Pz base

**Fig. 3:**
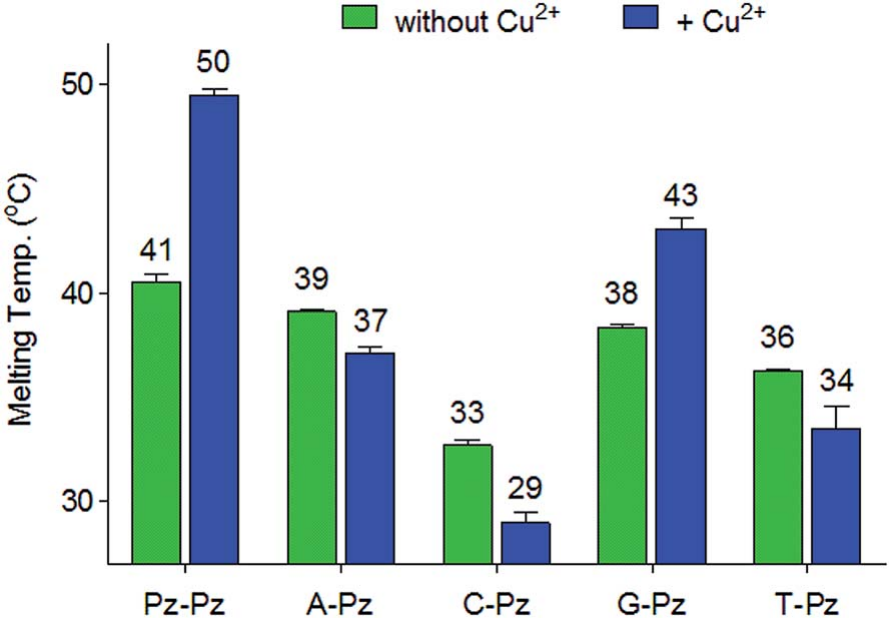
Melting temperatures of **1a** combined with different counter strands having A, G, C, T or Pz as the counter base. Conditions: 150 mM NaCl, 10 mM CHES buffer pH 9.0, 1 μM oligonucleotide, with or without 1 μM Cu^2+^, final volume of 200 μL.

Next, the selectivity of the new Pz ligandoside self pair was studied. Melting profiles of a Pz base containing strand **1a** with a complementary strand with a canonical base at the opposite position were measured at pH 9.0 (Fig. 3). The results showed that the Pz–C situation destabilizes the duplex dramatically while Pz–A/G/T arrangements give duplexes with lower but similar stability. In the presence of copper ions, the Pz–Cu–Pz metal base pair is by far the most stable structure which is the basis for selective Cu^2+^-complexation.

### Multiple metal ion binding by (Pz–Pz)_*n*_–DNA

To answer the question of whether the Pz ligandoside allows construction of DNA structures containing more than one metal ion stacking on top of each other we prepared oligonucleotides with several consecutive Pz ligandosides (Table 1, duplexes **3a**/**3b** and **4a**/**4b**).

**Fig. 4:**
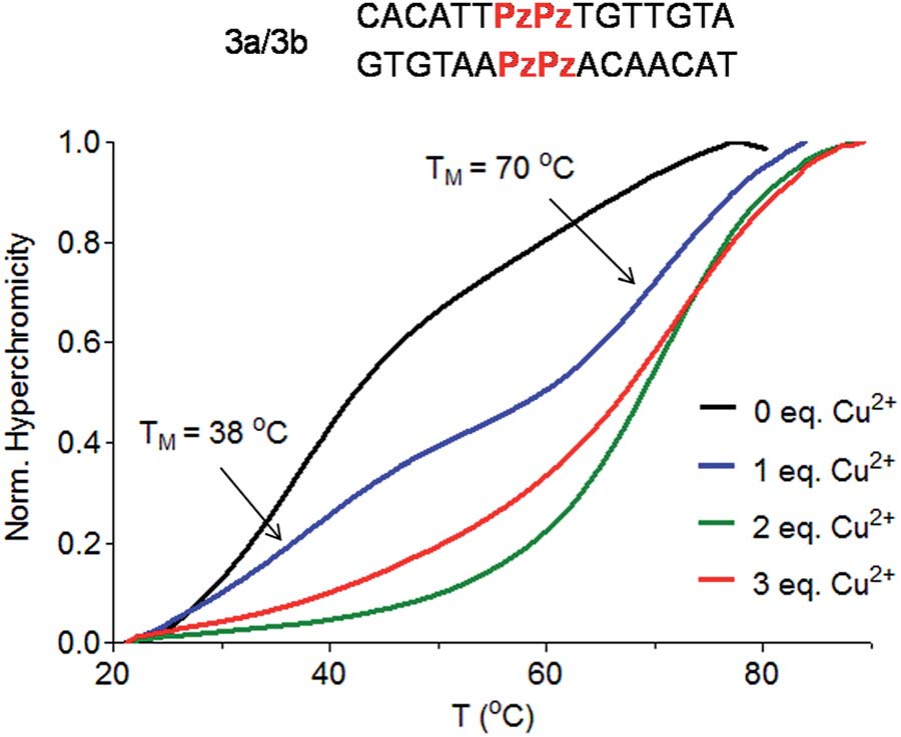
Schematic depiction of the investigated duplex **3a**/**3b** and Cu^2+^ titration melting profiles of duplex **3a**/**3b** (Pz = Pyrazole). Conditions: 150 mM NaCl, 10 mM CHES buffer pH 9.0, 1 μM oligonucleotide, final volume of 200 μL.

As a first approach, two complementary DNA single strands, each containing two neighbouring Pz pyrazole nucleosides, were hybridized to form the duplex **3a**/**3b**. Titration melting profiles are depicted in Fig. 4. In the absence of copper ions, the *T*_M_ of the duplex is decreased by about 11 °C (*T*_M_ = 38 °C, Fig. 4, black line) with respect to the double strand **0a**/**0b** and by 3 °C with respect to the duplex **1a**/**1b** with only one artificial Pz base pair.

When one equivalent of Cu^2+^ was added, the system showed two sigmoidal transitions (Fig. 4, blue line). One transition was detectable at *T*_M_ = 38 °C (corresponding to the *T*_M_ for the duplex without Cu^2+^) while the second transition appeared at a *T*_M_ of 70 °C (see also Fig. S6†).

These data indicate the presence of two well-defined species in solution. With one equivalent of Cu^2+^ ions in solution, half of the duplexes contains two metal ions (*T*_M_ = 70 °C), while the other duplexes feature no metal ion (*T*_M_ = 38 °C), arguing that Cu^2+^ complexation by the Pz ligandoside is a cooperative effect. This is in agreement with the observation of isosbestic points in the UV and CD titration experiments (Fig. S7 and S8†). When two or more equivalents of Cu^2+^ are present, the melting curves exhibit only the transition at 70 °C (Fig. 4, green and red lines), showing full saturation of all Cu^2+^ binding sites.

In order to investigate if more than two Cu^2+^ ions can be arranged in a line, we next studied a duplex with ten consecutive Pz ligandosides, where GC rich DNA sequences were used as the terminus (Table 1, duplex **4a**/**4b**).

**Fig. 5:**
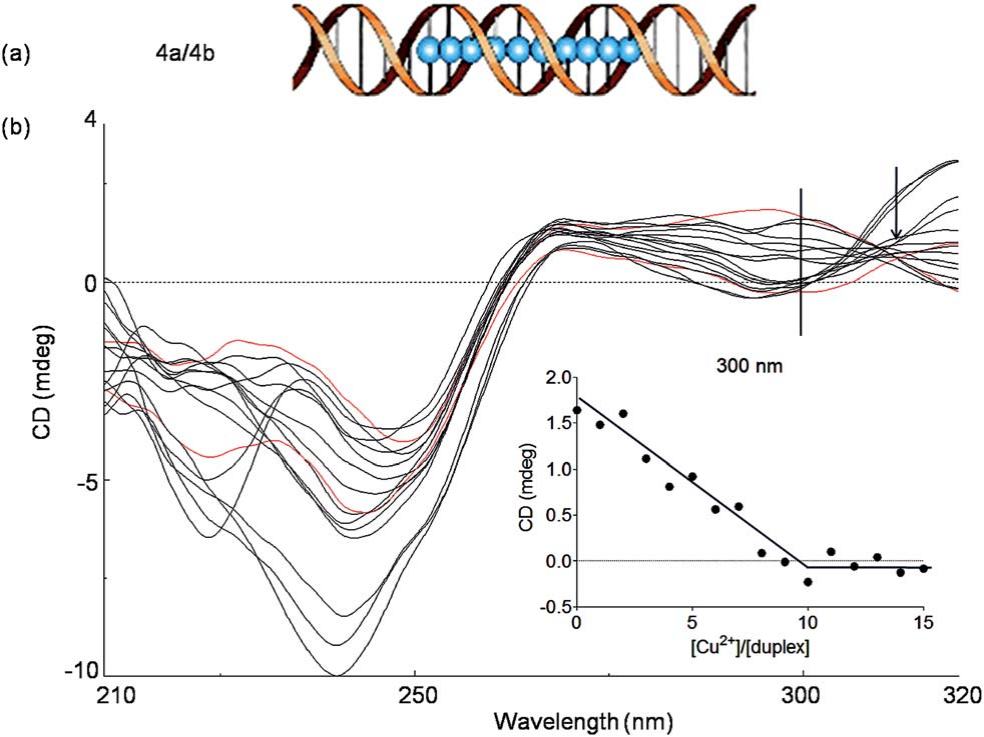
(a) Schematic depiction of the assembly of 10 Cu^2+^ pyrazole ligandosides inside a DNA duplex **4a**/**4b**; (b) CD spectral changes of the duplex **4a**/**4b** at various concentrations of Cu^2+^ (from 0 to 15 eq., step of 1 eq.), spectra of 0 eq. and 10 eq. are showed in red. Inset: plot of circular dichroic changes at 300 nm against the ratio of [Cu^2+^]/[**4a**/**4b**]. Conditions: 150 mM NaCl, 10 mM CHES buffer pH 9.0, 1 μM oligonucleotide, final volume of 200 μL.

After hybridization in the presence of Cu^2+^, the duplex **4a**/**4b** was again subjected to thermal UV analysis. However, due to its high stability, the melting point could not be accurately measured any more under the tested conditions. Therefore, CD titrations (Fig. 5, see also Fig. S9† for titrations at 240 nm) and UV titrations (Fig. S10†) at the same concentration were employed to monitor the Cu^2+^ assembling process. The overlaid CD spectra revealed that the structure of the duplex changes significantly upon increasing Cu^2+^ complexation. A true isosbestic point is not present any more. Plotting of the ellipticity at 300 nm against the equivalents of Cu^2+^ ions displayed, however, a linearly decrease until a [Cu^2+^]/[duplex] ratio of about 10 : 1 was reached, in agreement with the expected complexation stoichiometry. From the experiments we conclude that the assembly of 10 × (Pz–Cu–Pz) is indeed possible. However, the duplex forms a distorted structure that appears to be tolerated by the flexible Pz-self base pair. The complexation process is certainly not a cooperative event, which explains the absence of clear isosbestic point. We also observe at 240 nm structural changes beyond the titration of 10 eq. of Cu^2+^. We believe that these changes are due to additional association of Cu^2+^ with the multiple Pz-structure. These additional structural changes are not observable at longer wavelength. We therefore examined next how the presence of stiff, while crosslinking, salen ligandosides (S, Fig. 1a) would influence the copper coordination process.

### Multiple metal ion binding by hybrid Pz/S containing DNA

To this end mixed strands were prepared. For an initial study, oligonucleotides containing two salen nucleobases (S) and one additional Pz nucleobase in the middle were synthesized. In principle, this duplex can complex three metal ions (Table 1, duplex **5a**/**5b**).

**Fig. 6:**
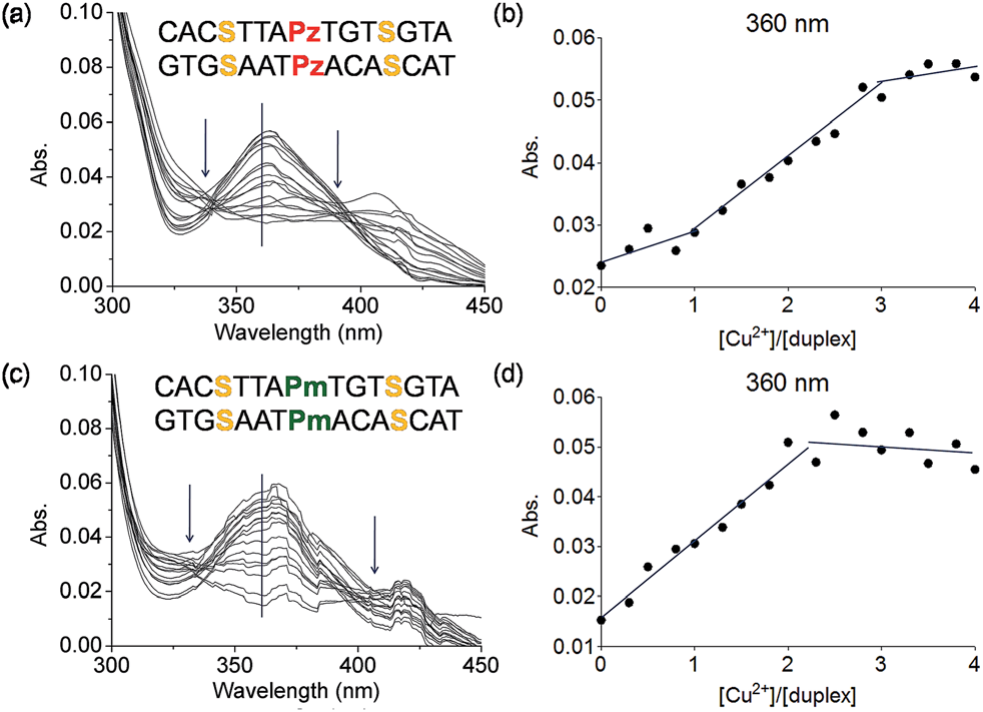
(a) Overlaid UV spectrum obtained at different concentrations of Cu^2+^ (from 0 to 4 eq.) of the duplex **5a**/**5b** and (b) plot of UV absorbance at 360 nm against the ration of [Cu^2+^]/[**5a**/**5b**]; (c) overlaid UV spectrum at different concentrations of Cu^2+^ (from 0 to 4 eq.) of the duplex **6a**/**6b** and (d) plot of UV absorbance at 360 nm against the ration of [Cu^2+^]/[**6a**/**6b**]. Conditions: 150 mM NaCl, 10 mM CHES buffer pH 9.0, 3 μM oligonucleotide, 30 eq. ethylenediamine, final volume of 200 μL.

The characteristic changes in the UV/Vis and CD spectrum of duplex **5a**/**5b** that occur upon titration with Cu^2+^ ions are presented in Fig. 6a, S11 and S12.† The overlaid curves show now isosbestic points again at *λ* = 340 and 396 nm. A plot of the absorbance at 360 nm against the equivalents of Cu^2+^ ions is depicted in Fig. 6b. Initially, when one equivalent of Cu^2+^ was added, a value of 0.025 for the absorbance was measured. With increasing amounts of Cu^2+^ (up to three equivalents), the absorbance raised to 0.055, which is a typical behaviour for copper complexation by a salen ligand.^42^ Further titration did not affect the absorbance any more. A similar trend was observed at 235 nm (Fig. S13†). These results confirm the complexation of three metal ions and the data reflect that the first metal ion is complexed by the Pz–Pz pair, followed by complexation of the other two ions by the two salen ligandosides. It is clear that the complexation process by both ligandosides follows a different kinetic scheme.

More experimental support for this hypothesis was obtained when the Pz nucleoside was replaced by methylated Pz (Pm), which is unable to coordinate copper ions. UV titration of duplex **6a**/**6b** containing now the Pm instead of the Pz ligandosides showed that the absorbance at 360 nm increased up to a value of 0.050 after addition of 2 equivalents of Cu^2+^, characteristic for salen copper complexes (Fig. 6c/d). Further addition of metal ions didn't change the absorbance. As Pm fails to coordinate copper, the ions go directly into the two salen base pairs. Because the increase at 360 nm for **6a**/**6b** is similar to those for **5a**/**5b**, we conclude that two equivalents of Cu^2+^ are complexed in this case. These observations support the hypothesis, that the Pz–Pz base pair is the first ligandoside loaded with a metal ion.

**Fig. 7:**
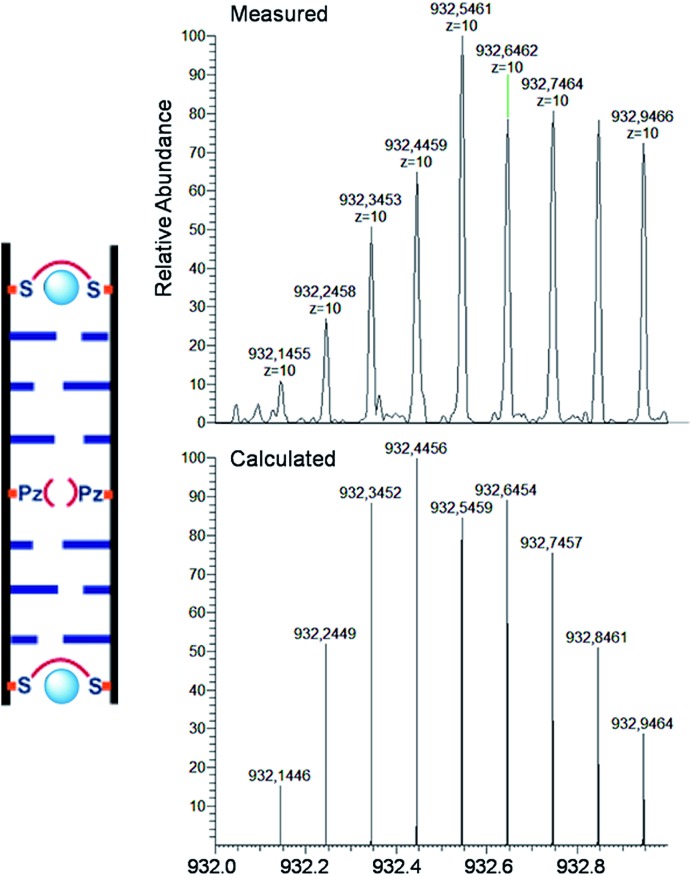
ESI-Mass spectrum and comparison of experimental data with calculated molecular weights of duplex **6a**/**6b** with 2 eq. Cu^2+^, molecular formula C_316_H_384_O_176_N_96_P_28_Cu_2_. Peaks contain 10 charges (top) compared with calculation (bottom). Conditions: 150 mM NH_4_OAc, 30 μM oligonucleotide, 30 eq. ethylenediamine.

For the characterization of these duplexes, ESI-Mass measurements were performed. Double strand **1a**/**1b**, hybridized with copper, provided always two main signals corresponding to the two single strands. We were unable to detect the desired mass peaks of the duplex containing a copper ion inside, confirming that complexation and decomplexation of Pz–Pz ⇆ Pz–Cu–Pz is fast. In contrast, the same experiment performed with the duplex **5a**/**5b** with an excess of ethylenediamine and 3 equivalents of Cu^2+^ showed in the mass spectrum quantitative formation of the duplex containing two copper complexes (Fig. 7 and S14†). The third copper ion could not be detected in agreement with fast Pz–Cu–Pz decomplexation. Obviously, and in agreement with earlier data,^42^ in the MS experiment, only the Cu–salen complexes are stable enough to survive the ESI conditions.

**Fig. 8:**
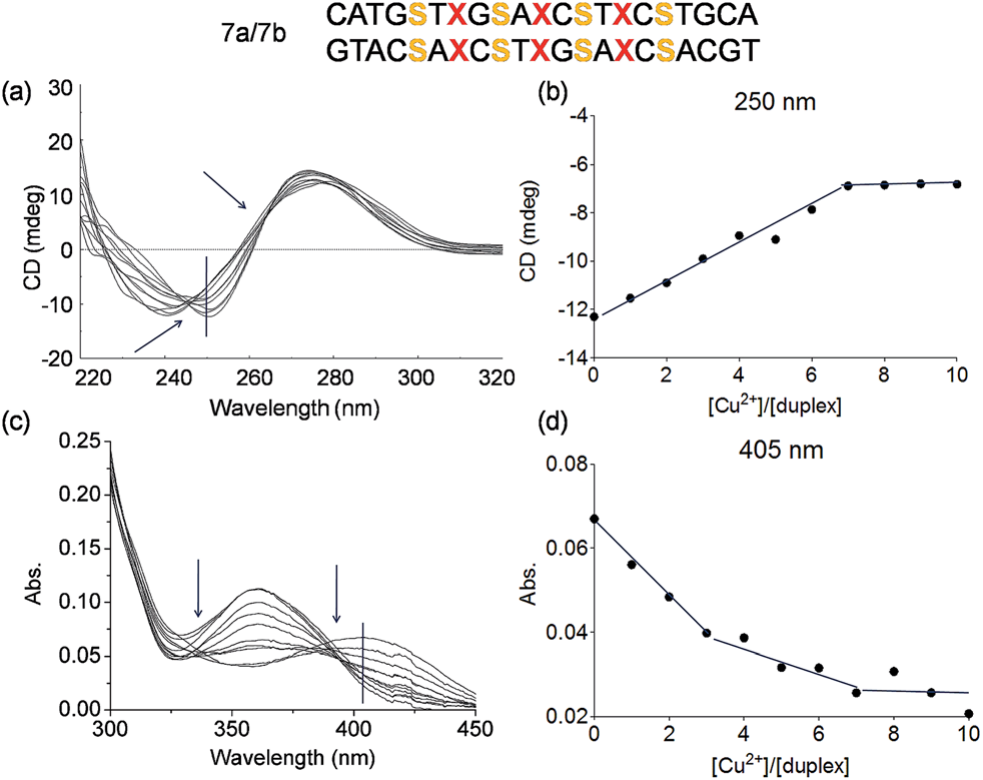
Schematic depiction of the investigated duplex, S = salen, X = pyrazole. (a) Overlaid CD spectrum at different concentrations of Cu^2+^ (from 0 to 10 eq.) of the duplex **7a**/**7b**; (b) plot of circular dichroic changes at 250 nm against the ration of [Cu^2+^]/[**7a**/**7b**]; (c) overlaid UV spectrum under the same conditions as in (a); (d) plot of UV absorbance at 405 nm against the ration of [Cu^2+^]/[**7a**/**7b**]. Conditions: 150 mM NaCl, 10 mM CHES buffer pH 9.0, 3 μM oligonucleotide, 30 eq. ethylenediamine, final volume of 200 μL.

A more complicated scenario was found when four Pz pairs were mixed with three salen pairs in duplex **7a**/**7b**. The complexation of copper ions in this new situation was confirmed by CD titration. The CD data prove complexation of 7 equivalents of Cu^2+^ ions within the duplex (Fig. 8a/b; see also Fig. S15† for overlaid UV spectrum). The overlaid CD spectrum shows, despite the complex ligandoside composition of the duplex, two isosbestic points at 246 nm and 265 nm arguing for cooperative metal complexation. A plot of the UV absorbance at 405 nm during metal ion titration is displayed in Fig. 8d (see also Fig. S16† for absorbance at 360 nm). The UV data reveal two different trends which correspond to the two different coordination events established by the Pz and the salen base. Addition of three equivalents of Cu^2+^ led to a first rather sharp decrease in the absorbance at 405 nm, which corresponds to the first coordination process. With 3 Pz ligandosides we assume again initial complexation of Cu^2+^ by this ligandoside system. Thereafter, addition of another four equivalents of Cu^2+^ gave a further change of the UV data. Now, the reduction of the absorbance is slower. With all these evidences in hand, and accordingly to the previous hypothesis and results, we conclude that the Pz pairs complex the copper ions first while the 4 salen ligandosides are filled in the second process.

### Catalysis of a Diels–Alder reaction by (Pz–Cu^2+^–Pz)_*n*_–DNA

In order to investigate if the multiple Cu-containing DNA strands are able to exhibit some function, *e.g.* as catalyst, we started to investigate their ability to catalyze a Diels–Alder reaction. For other enantioselective Diels Alder reactions based on Cu^2+^–ligand–DNA see ref. 47 and 48 and for Cu^2+^-G-quadruplex see ref. 49.

The D–A reaction between aza-chalcone (**1**) and cyclopentadiene (**2**), using duplex **8a**/**8b** (system with five Cu^2+^ ions complexed up in Pz containing DNA) as a catalyst was selected for this initial study and the results are summarized in Table 2. For us a central question was if the duplexes, which contain the metal ions in the middle are able to perform the catalysis and whether enantioselectivity can be obtained. We noted that both single and double strands with the Pz-ligands catalyze the reaction in the presence of Cu^2+^ and in both cases some chirality transfer is observed. At the same time, the complex accelerates the conversion. Copper counter ion and surrounding pH are also playing a role in the catalytic process. The result shows that the Pz-containing DNA strands can be turned into catalyst. Now, their efficacy and the ee-values obtained for the reaction need to be improved.

**Table 2:** Results of the catalytic Diels–Alder reaction with ds **8a**/**8b** ^*a*^

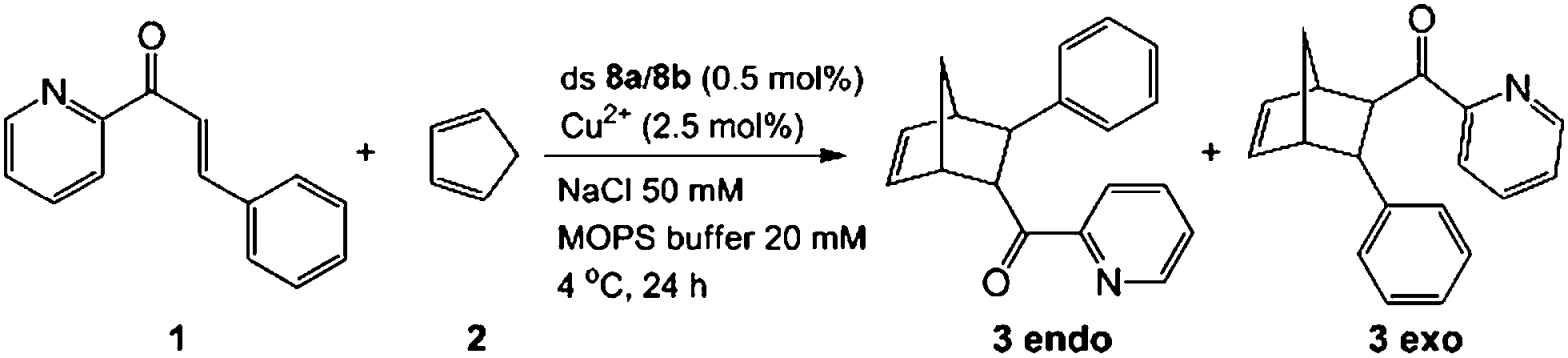						
Entry	Ligand	[Cu]	pH	ee (%)	Endo/exo	Conv. (%)
1	**8a**/**8b**	Cu(NO_3_)_2_	6.5	39	93	97
2	—	Cu(NO_3_)_2_	6.5	1	87	76
3	ss **8a**	Cu(NO_3_)_2_	6.5	29	93	96
4	**8a**/**8b**	Cu(NO_3_)_2_	7.4	36	86	79
5	**8a**/**8b**	Cu(NO_3_)_2_	9.0	23	80	47
6	**8a**/**8b**	CuSO_4_	6.5	32	87	52
7	**8a**/**8b**	Cu(OTf)_2_	6.5	29	84	46
8	**8a**/**8b**	—	6.5	3	76	19

^*a*^See ESI for reaction details. All data are averaged over two experiments. OTf = trifluoromethanesulfonate.

## Conclusion

The metal base pair, pyrazole ligandoside, was synthesized and incorporated into oligonucleotides. The ligandoside system shows a pH-dependent complexation behaviour due to the need for deprotonation of the phenolic groups. When the OH groups are replaced by a methoxy moiety as in the Pm ligandoside, no metal ion coordination is observed. Cu^2+^ ions stabilize a Pz–Pz containing duplex when compared to a canonical G–C pair, at pH 9.0.

The Pz–Pz ligandoside base pair allows complexation of up to 10 Cu^2+^ ions within a duplex. Better complexation of multiple Cu^2+^ ions is, however, observed when the unbridged Pz ligandoside is combined with the bridged salen (S) system which seems to add so much integrity and duplex stiffness that the metal ion complexation process is dominated by cooperative effects. The copper ions show a kinetic preference for complexing first into the Pz–Pz base pair while the salen complex is loaded in a second independent step. This stepwise complexation enables in principle the design of logic gates. The described complexation properties are indeed now the basis for the construction of defined metal ions clusters within oligonucleotides, with the goal to construct multi-metal ion based catalysts and logic gates.
