# Supplementary material for: DNA based multi-copper ions assembly using combined pyrazole and salen ligandosides
Source: Chem Sci. 2014 Aug 11;6(1):632–8. doi: 10.1039/c4sc01567c (PMC5588782; doi:10.1039/c4sc01567c)
Supplement: Supplementary file 1 [file SC-006-C4SC01567C-s001.pdf]

Electronic Supplementary Material (ESI) for Chemical Science.
This journal is © The Royal Society of Chemistry 2014

Electronic Supplementary Information

DNA Based Multi-Copper Ions Assembly using Combined Pyrazole and Salen Ligandosides

M. Su,[a] M. Tomás-Gamasa,[a] and T. Carell[a]

Center for Integrated Protein Science at the Department of Chemistry,

Ludwig-Maximilians Universität München, Butenandtstr. 5-13, 81377 Munich

E-mail: thomas.carell@cup.uni-muechen.de

Table of contents

I. General method for oligonucleotide synthesis and characterization ……………….....S2

II. Selected thermodynamic data and spectroscopy data for melting curves and CD experiments …………………..……………………………………………...S5

III. Typical procedure and supplementary data of Diels-Alder reaction using pyrazole ligandoside based catalyst …………………………………………….…………….S16

I. General method for oligonucleotide synthesis and characterization

Oligonucleotide synthesis

DNA Oligonucleotide synthesis was performed on an Applied Biosystems Incorporated 394 automated synthesizer. Phosphoramidites and LV-PS (Low Volume Polystyrene) columns were purchased from *Glen Research Corporation*. All oligodeoxynucleotides were synthesized on a 200 nmol scale with standard DNA synthesis cycles (trityl off mode). For the artificial monomers, the coupling time was extended to 600 s. The efficiency of oligomers synthesis was monitored by trityl absorbance. Cleavage from the solid support and deprotection were achieved by incubation in a 28% ammonium hydroxide solution and 40% mono-methylamine solution (1/1) at 65 °C for 10 min.

The solvents were removed in a *Speed Vac* concentrator and the pellet redissolved in Milli-Q water. Semi-preparative HPLC was achieved using a *Macherey-Nagel* C18 column (5 mm, 9.4×250 mm) on *Waters* Breeze 2487 Dual λ Array Detector, 1525 Binary HPLC Pump.

Conditions:

Buffer A, 0.1 M TEAA.

Buffer B, 0.1 M TEAA in 80% acetonitrile.

Started at 0% B; linear gradient to 30% B (or 40% B) over 45 min, flow rate: 5 mL/min.

The purified fractions were concentrated, desalted on *Waters* Sepac-C18 cartridges and concentrated again. The obtained oligomers were characterized by Matrix Assisted Laser Desorption Ionization Time of Flight (MALDI-TOF) on Bruker Daltonics Autoflex II. Concentration of oligonucleotide solutions was calculated from the UV absorbance at 260 nm on a Nanodrop ND-1000 spectrophotometer with the following molar extinction coefficients: ε(Pz) = 7600, ε(Pm) = 5685, ε(A) = 15200, ε(G) = 12000, ε(C) = 7100, ε(T) = 8400.

Sample preparation and melting experiments

Melting profiles were measured on a JASCO V-650 spectrometer using quartz glass cuvettes with 10.00 mm path length. The samples contained 150 mM NaCl, 10 mM CHES buffer and 1 μM of each strand in a final volume of 200 μL. For strands **1a**/**1b**, **2a**/**2b**, **3a**/**3b**, and **4a**/**4b** the oligonucleotides with Cu^{2+} were hybridized by slowly cooling down the samples from 90 °C to room temperature overnight. Each sample was prepared and measured at least three times in parallel. The solutions were covered with silicon oil and tightly plugged. UV Absorbance was recorded in the forward and reverse direction at temperatures from 20 °C to 80 °C with a slope of 1 °C/min. Three denaturing and renaturing ramps were performed for each sample and averaged for evaluation of the melting point. T_M values were calculated as the zero-crossing of 2nd derivate of the 349 nm background corrected change in hyperchromicity at 260 nm.

UV / CD spectra measurements

UV and CD spectra were measured on a JASCO V-650 or J-810 spectrometer using quartz glass cuvettes with 10.00 mm path length. For the strands with mixed salen and pyrazole bases, **5a**/**5b**, **6a**/**6b** and **7a**/**7b**, the duplex (3 μM of each strand) was first reannealed in 150 mM NaCl, 10 mM CHES buffer without Cu^{2+} ions or ethylenediamine overnight. Then an excess of ~30 fold ethylenediamine, diluted in degased Milli-Q water, was added and the solution was incubated at 4 °C overnight. Finally, different amounts of Cu^{2+} were added into individual samples and final volume was constant (200 μL). After Cu^{2+} was added, the samples

were kept at 4 °C and measured in 2 h. Each sample was prepared and measured at least twice in parallel at 20 °C. For UV measurements, five spectra were accumulated. For CD measurements, ten spectra from 320 nm to 210 nm were accumulated with scanning speed of 500 nm/min at 20 °C. Blank correction was made of aqueous solution of buffer and salt and measured for each series of titration measurement.

ESI Mass measurements

For duplex **5a**/**5b**, 30 μM of duplex was hybridized in 150 mM NH_4OAc as above. Here, no CHES buffer and NaCl aq. were used. After addition of ethylenediamine, the mixture was incubated at 4 °C overnight, then Cu^{2+} (3 eq.) was added. Before ESI measurement, a mixture (10 μL) of imidazole (250 mM) and piperidine (250 mM) in 80 % aq. acetonitrile was added to the sample (40 μL).

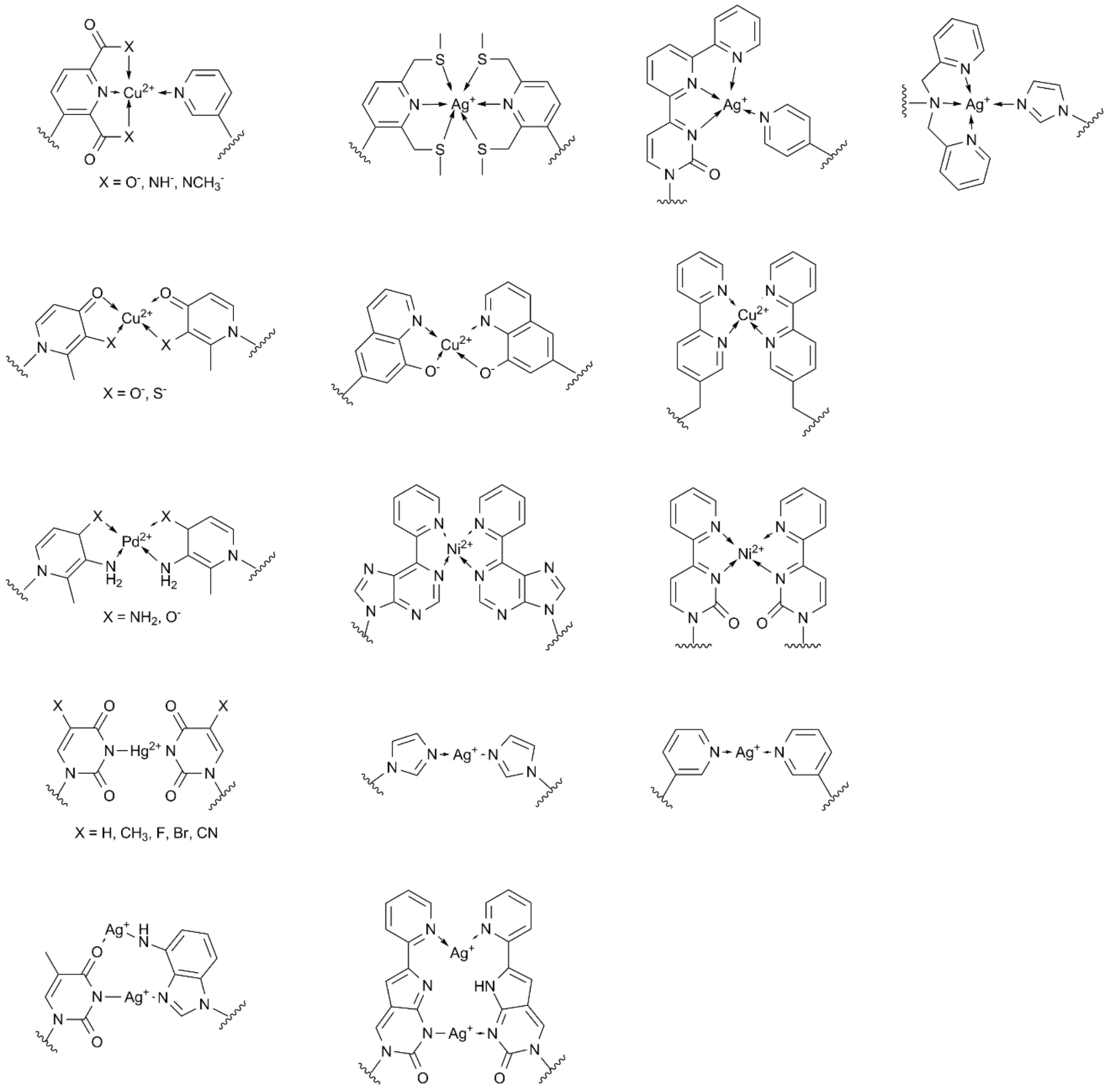

Fig. S1 Summary of reported metal base pairs. Backbone part is not shown here and only one kind of metal ion is shown.

II. Selected thermodynamic data and spectroscopy data for melting curves and CD experiments

Table S1 Thermodynamic data for duplex **1a**/**1b** [a]

pH	- Cu^{2+} ΔH° [kcal mol^{-1}]	- Cu^{2+} ΔS° [cal K^{-1}mol^{-1}]	- Cu^{2+} ΔG°$_{310K}$ [kcal mol^{-1}]	+ Cu^{2+} ΔH° [kcal mol^{-1}]	+ Cu^{2+} ΔS° [cal K^{-1}mol^{-1}]	+ Cu^{2+} ΔG°$_{310K}$ [kcal mol^{-1}]
6.0	-119.3	-373	-3.7	-131.9	-414	-3.6
7.4	-128.5	-397	-5.4	-125.9	-390	-4.8
9.0	-121.5	-388	-1.1	-111.0	-345	-4.0

[a] These values were determined by van't Hoff plots from the melting profiles.

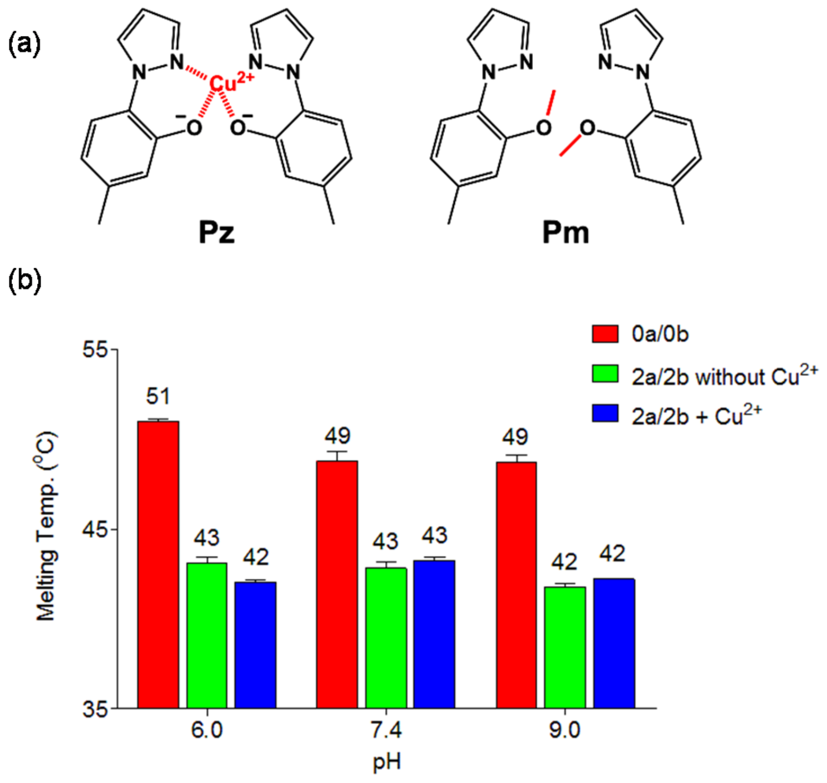

Fig. S2 (a) Depiction of the Pz-Pz and the Pm-Pm base pairs; (b) Melting temperatures of duplex **2a**/**2b** at different pH. Conditions: 150 mM NaCl, 10 mM Na_2HPO_4/NaH_2PO_4 buffer pH 6.0 / 7.4 or CHES buffer pH 9.0, 1 μM oligonucleotide, with or without 1 μM Cu^{2+}, final volume of 200 μL.

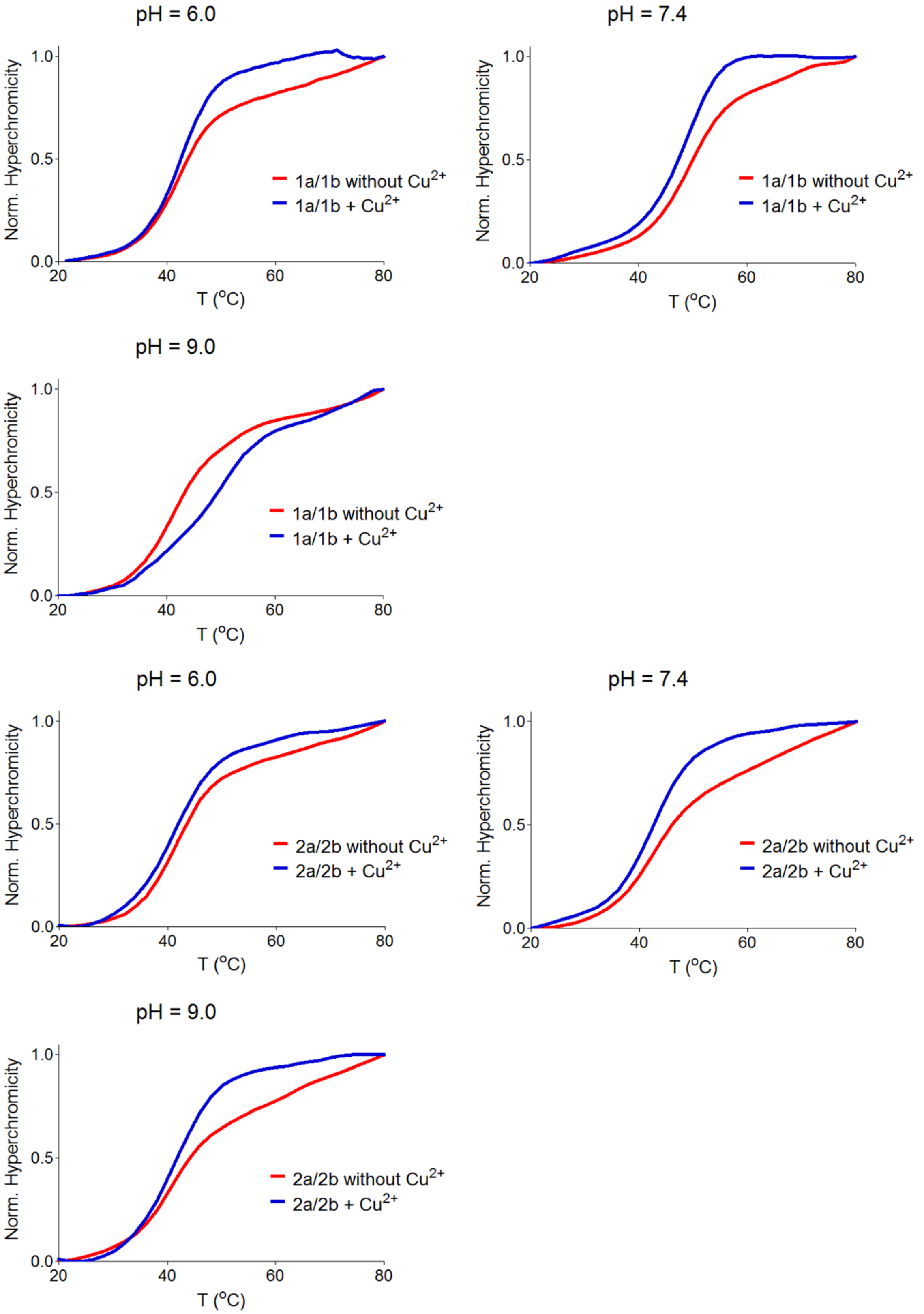

Fig. S3 Melting curves of duplexes **1a**/**1b** and **2a**/**2b** at different pH. Conditions: 150 mM NaCl, 10 mM Na_2HPO_4/NaH_2PO_4 buffer pH 6.0 / 7.4 or 10 mM CHES buffer pH 9.0, 1 μM oligonucleotide, with or without 1 μM Cu^{2+}, final volume of 200 μL.

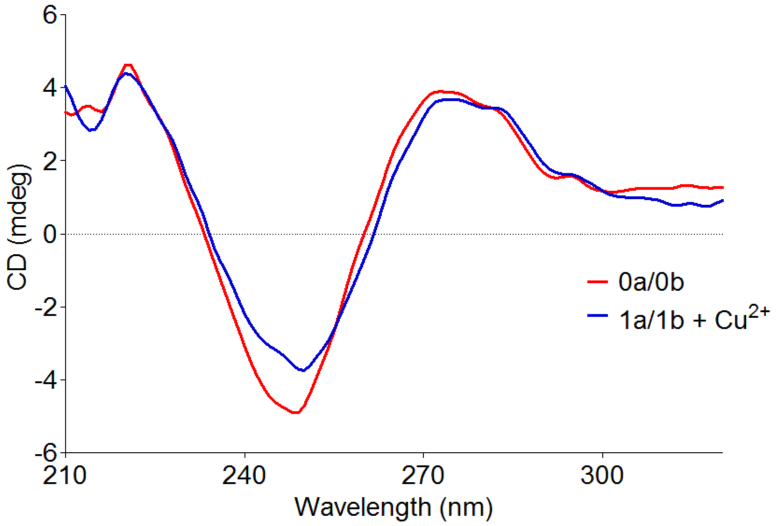

Fig. S4 CD spectrum of duplex **1a**/**1b** with equal equivalents of Cu^{2+}, compared to canonical duplex **0a**/**0b**. Conditions: 150 mM NaCl, 10 mM CHES buffer pH 9.0, 1 µM oligonucleotide, 1 µM Cu^{2+}/Mn^{3+}, final volume of 200 µL, 20 °C.

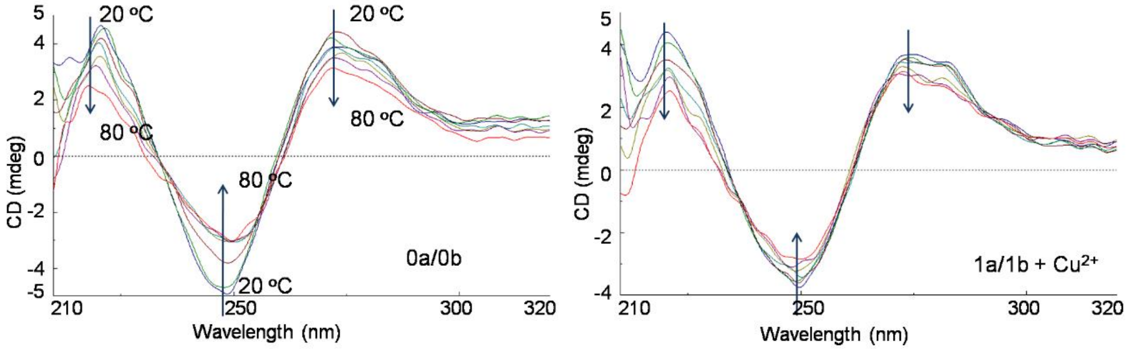

Fig. S5 Overlaid CD spectrum of duplex **1a**/**1b** with equal equivalents of Cu^{2+}, compared to canonical duplex from 20 °C to 80 °C. Conditions: 150 mM NaCl, 10 mM CHES buffer pH 9.0, 1 µM oligonucleotide, 1 µM Cu^{2+}, final volume of 200 µL.

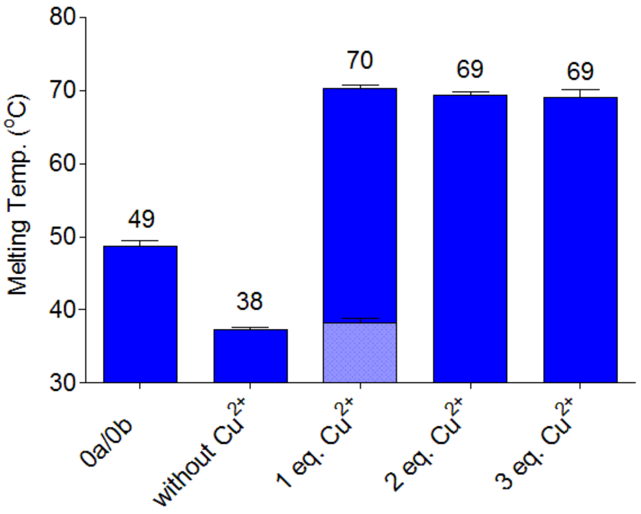

Fig. S6 Melting temperatures of duplex **3a**/**3b** with 0 to 3 eq. Cu^{2+}. Conditions: 150 mM NaCl, 10 mM CHES buffer pH 9.0, 1 μM oligonucleotide, final volume of 200 μL.

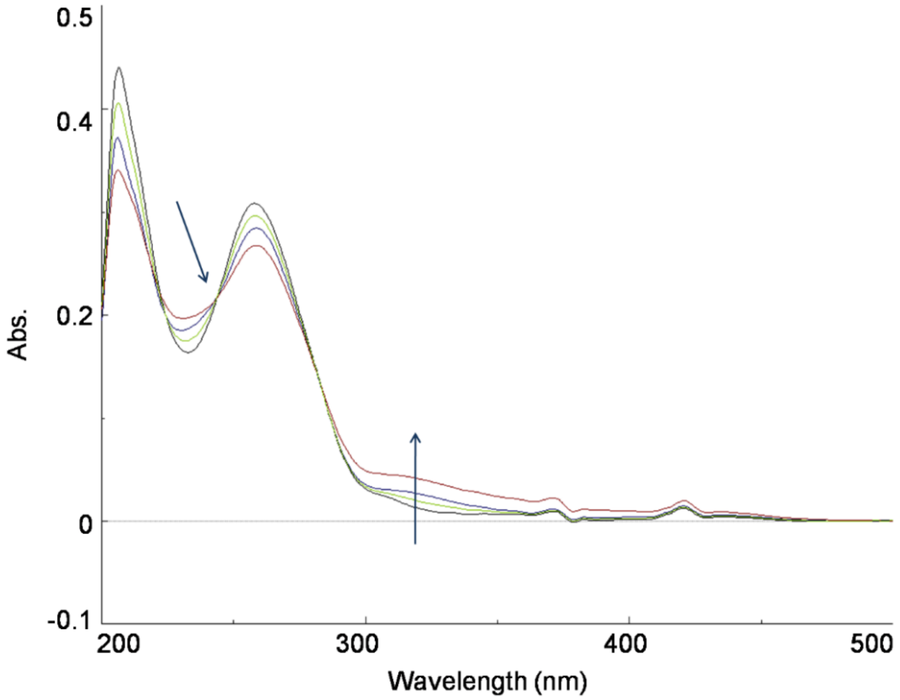

Fig. S7 Overlaid UV spectrum of duplex **3a**/**3b** at various concentrations of Cu^{2+} (from 0 to 3 eq., step of 1 eq.). Conditions: 150 mM NaCl, 10 mM CHES buffer pH 9.0, 1 μM oligonucleotide, final volume of 200 μL.

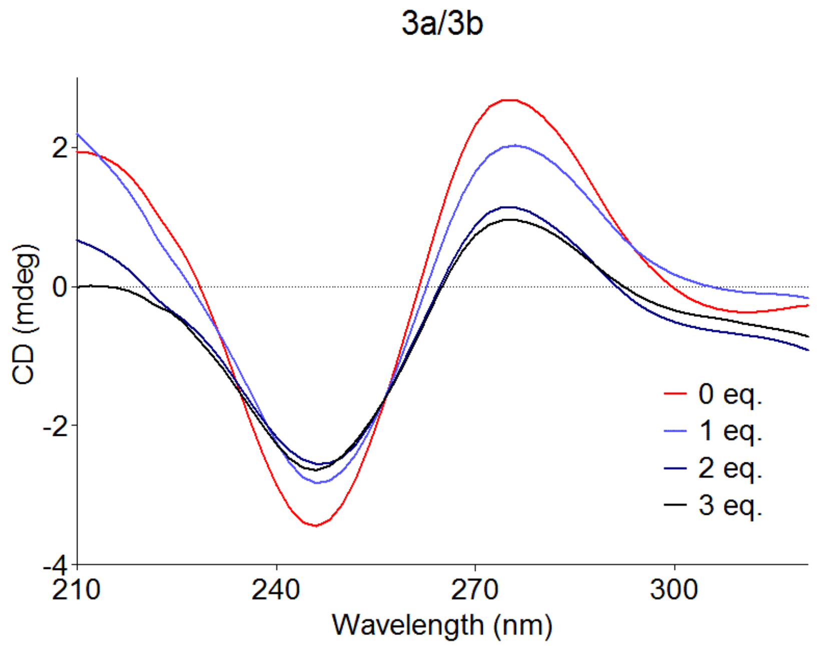

Fig. S8 Overlaid CD spectrum of duplex **3a**/**3b** at various concentrations of Cu^{2+} (from 0 to 3 eq., step of 1 eq.). Conditions: 150 nm NaCl, 10 mM CHES buffer pH 9.0, 1 µM oligonucleotide, final volume of 200 µL.

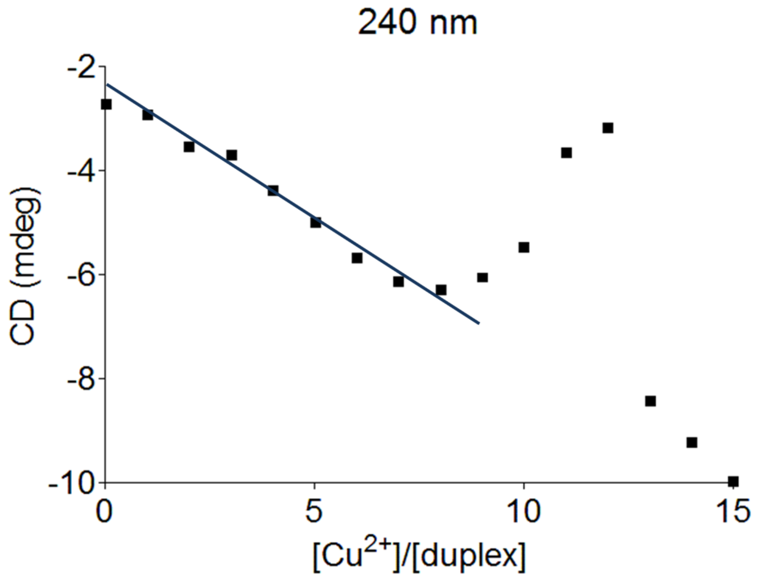

Fig. S9 Plot of CD spectral changes of the duplex at 240 nm of duplex **4a**/**4b** against the ration of [Cu^{2+}]/[**4a**/**4b**].

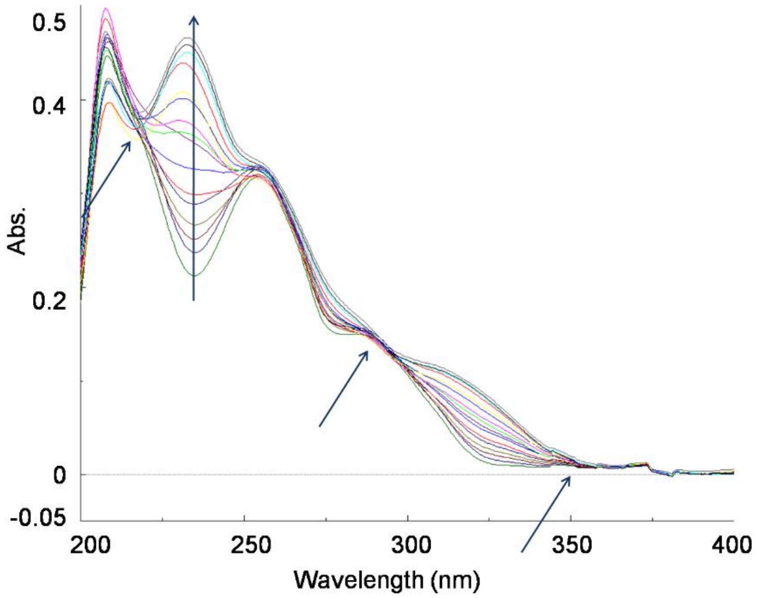

Fig. S10 Overlaid UV spectrum of duplex **4a**/**4b** at various concentrations of Cu^{2+} (from 0 to 15 eq., step of 1 eq.). Conditions: 150 mM NaCl, 10 mM CHES buffer pH 9.0, 1 μM oligonucleotide, final volume of 200 μL.

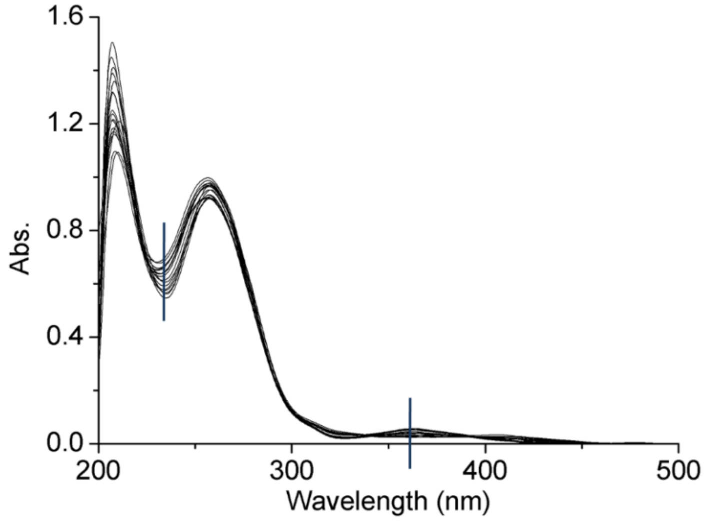

Fig. S11 Overall UV spectrum of duplex **5a**/**5b** at various concentrations of Cu^{2+} (from 0 to 4 eq.). Conditions: 150 nm NaCl, 10 mM CHES buffer pH 9.0, 3 µM oligonucleotide, final volume of 200 µL.

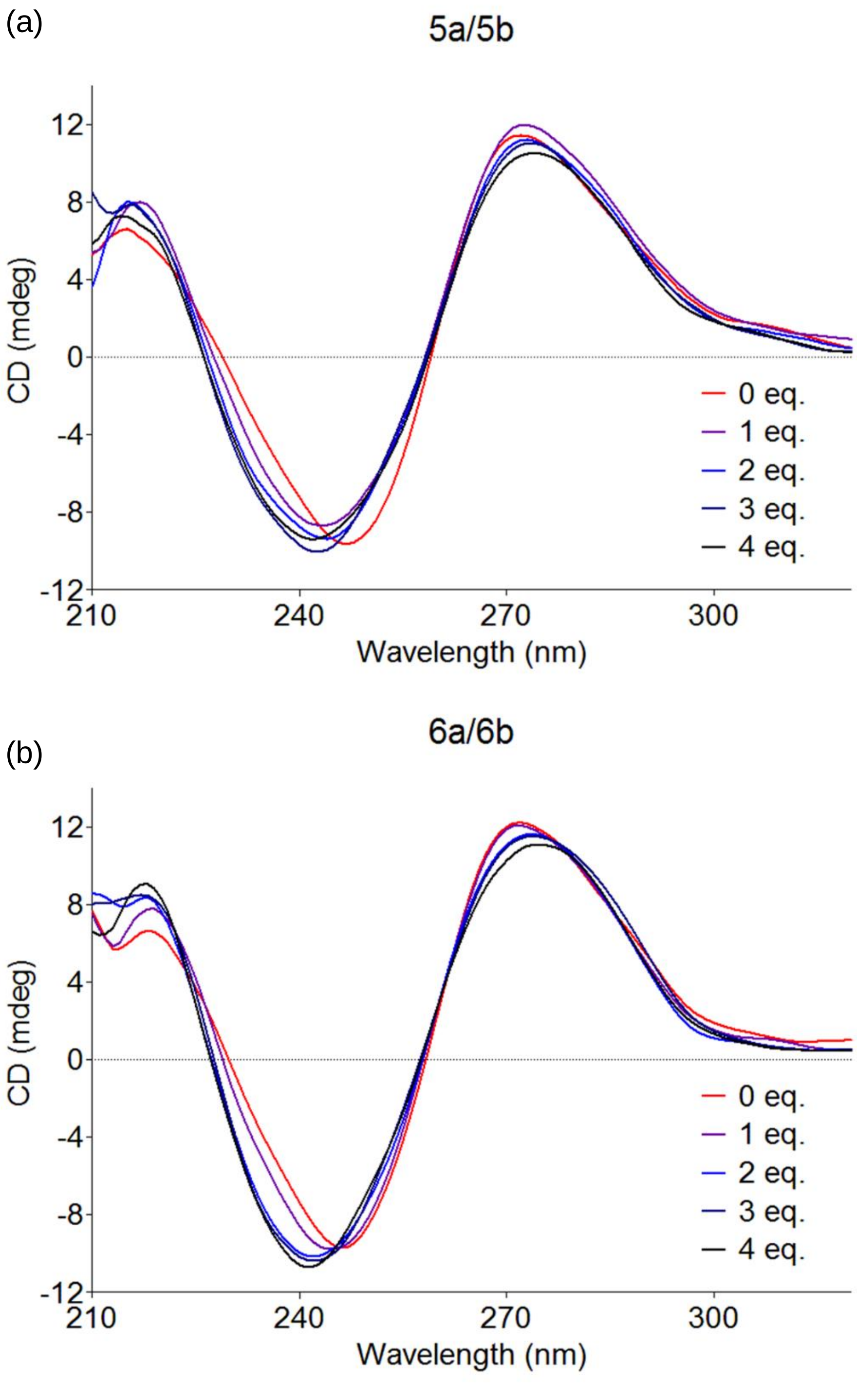

Fig. S12 Overlaid CD spectrum of (a) duplexes **5a**/**5b** and (b) **6a**/**6b** at various concentrations of Cu^{2+} (from 0 to 4 eq., step of 1 eq.). Conditions: 150 nm NaCl, 10 mM CHES buffer pH 9.0, 3 μM oligonucleotide, final volume of 200 μL.

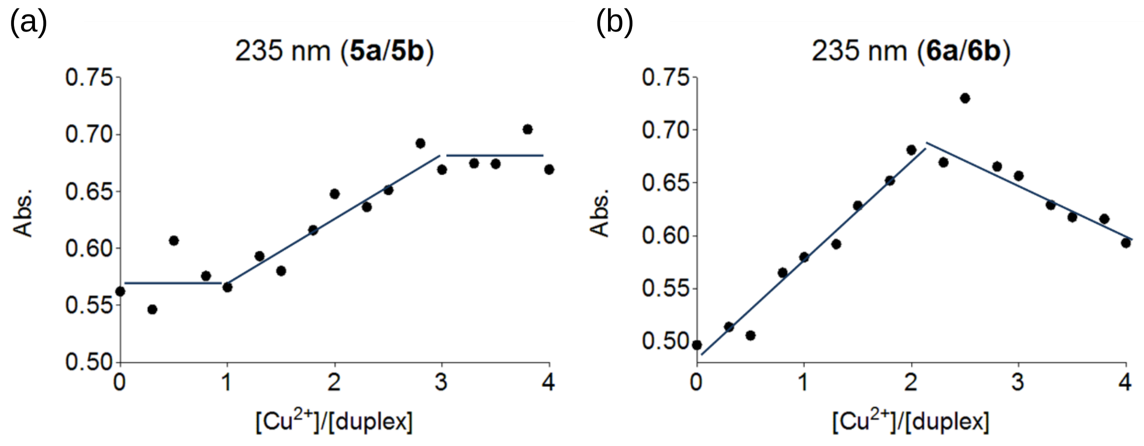

Fig. S13 Plot of UV absorbance at 235 nm of (a) duplex **5a**/**5b** and (b) **6a**/**6b** against the ration of [Cu^{2+}]/[**5a**/**5b**] and [Cu^{2+}]/[**6a**/**6b**].

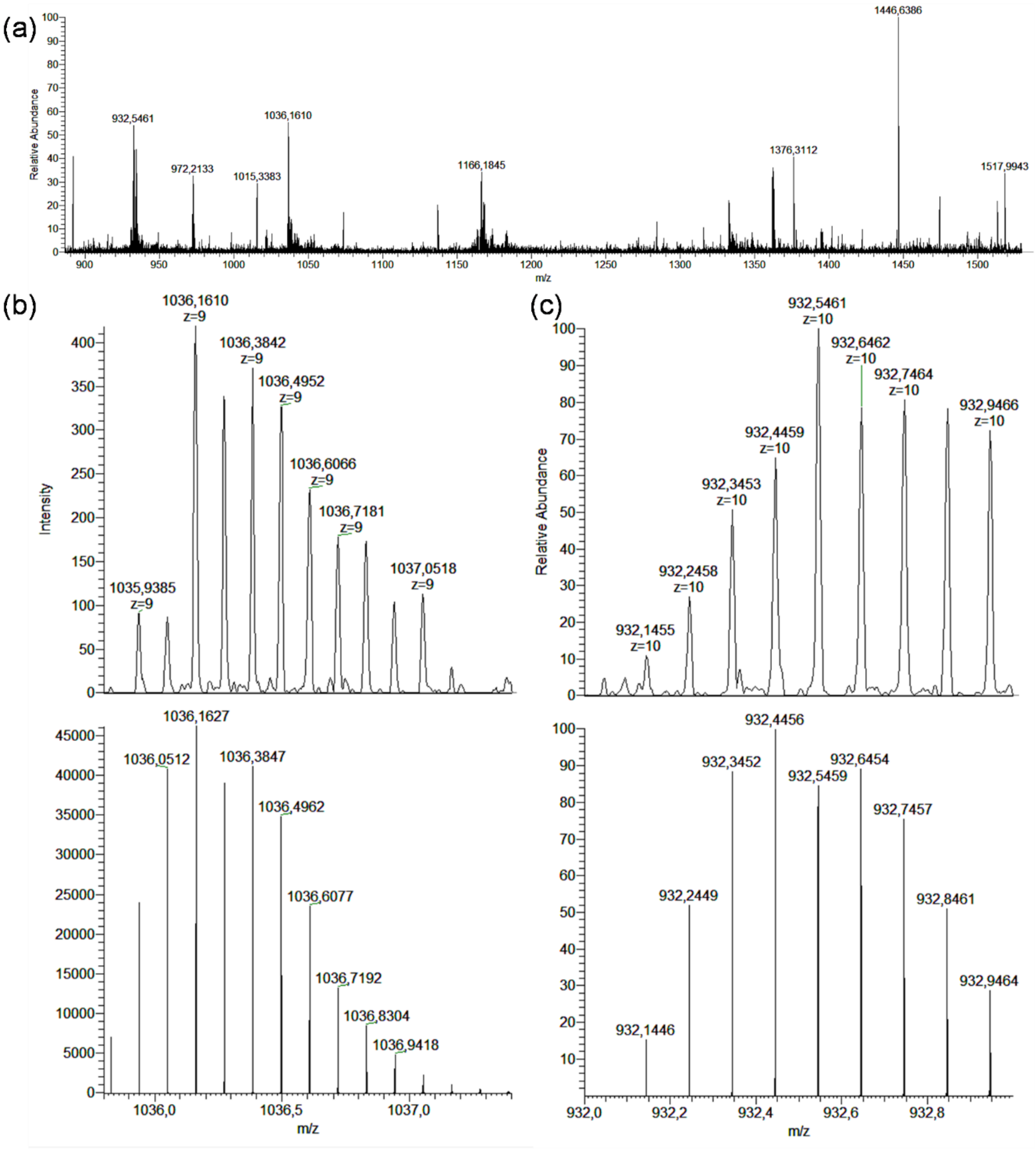

Fig. S14 ESI-Mass spectrum and comparison of experimental data with calculated molecular weigts of duplex **5a**/**5b** with2 eq. Cu^{2+}. (a) Overall ESI spectrum; (b) Peaks contain 9 charges (top) compared with calculation (bottom); (c) Peaks contain 10 charges (top) compared with calculation (bottom). Conditions: 150 mM NH_4OAc, 30 µM oligonucleotide, 30 eq. ethylenediamine.

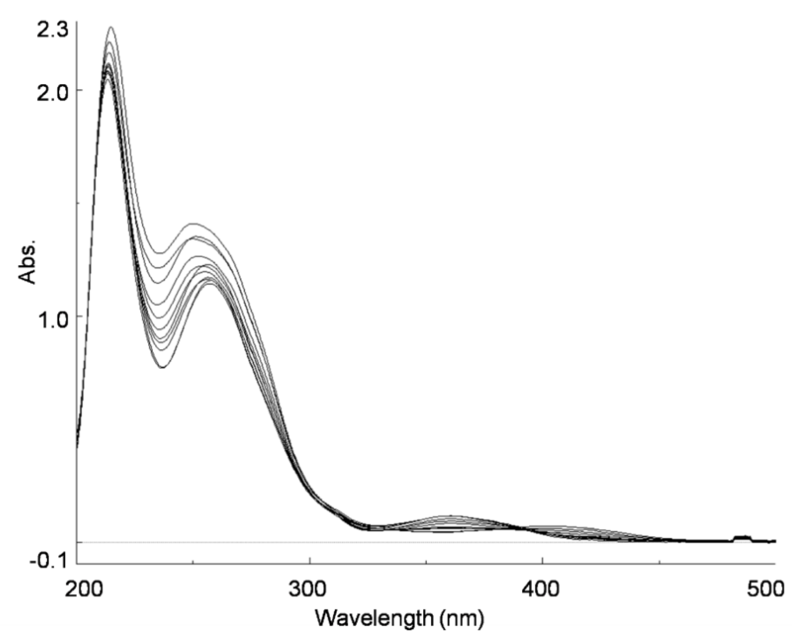

Fig. S15 Overall UV spectrum of duplex **7a**/**7b** at various concentrations of Cu^{2+} (from 0 to 10 eq.). Conditions: 150 nm NaCl, 10 mM CHES buffer pH 9.0, 3 µM oligonucleotide, final volume of 200 µL.

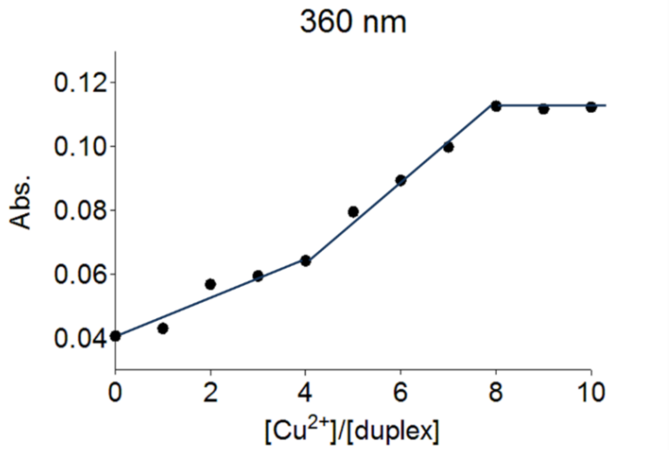

Fig. S16 Plot of UV absorbance at 360 nm of duplex **7a**/**7b** against the ration of [Cu^{2+}]/[**7a**/**7b**].

III. Typical procedure and supplementary data of Diels-Alder reaction using pyrazole ligandoside based catalyst

General method:

ODN **8a** and **8b** (5 nmol), copper(II) salts (1 mM, 25 µL, 25 nmol, 5 eq., 2.5 mol%), ammonium acetate aq. (pH = 9.0, 0.1 M, 30 µL), NaCl aq. (0.5 M, 100 µL) and Milli-Q water (145µL) were mixed to a final volume of 300 µL. The duplex was hybridized by heating at 90 °C for 5 min, then slowly cooled down to room temperature on a water bath. The solution was lyophilized to dryness and redissolved in MOPS buffer (pH = 6.5, 7.4 or 9.0, 20 mM, 1 mL). Dienophile **1**, which was prepared according to the procedure reported in *J. Am. Chem. Soc.* **1996**, *118*, 7702-7707, in acetonitrile (0.1 M, 10 µL, 1 µmol) was added. Cyclopentadiene **2**, freshly distilled from dicyclopentadiene (7.5 µL), was added to initiate the reaction. The mixture was shaken for 24 hours at 4 °C, followed by the extraction with diethyl ether (3 × 4 mL), dried over $MgSO_4$, filtered and concentrated. The crude product was dissolved in heptane and 2-propanol (v/v 99/1) and analyzed by chiral HPLC directly.

HPLC analysis:

Schimadzu CBM-20A communication bus module, SPD-M20A prominence diode array detector. *Daicel Chiralpak* IB 81325, 250 mm × 4.6 mm. Conditions: 1% 2-propanol in heptanes, 0.5 mL/min, column temperature 40 °C, detected from 190 nm to 500 nm, integration at 212 nm. Conversion of **1** was calculated using the following formula:

Conversion of **1**(%) = $PA_3 / (PA_3 + PA_1 / f)$ (PA: peak area)

where PA_1 and PA_3 are the HPLC peak areas of **1** and **3**, respectively, and f is the correction factor determined to be 0.69 from the fitting curve (Fig. S17).

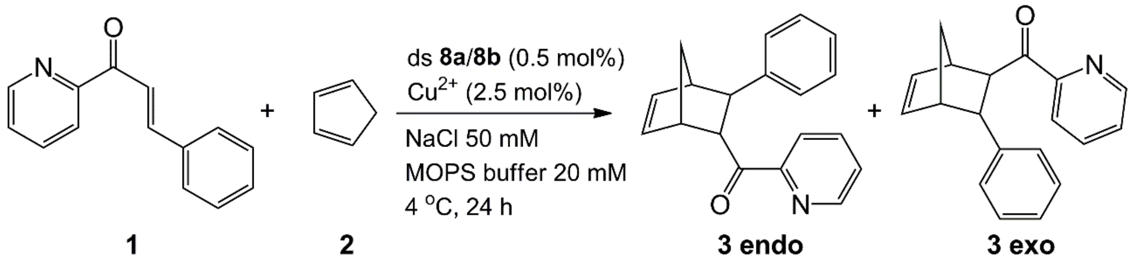

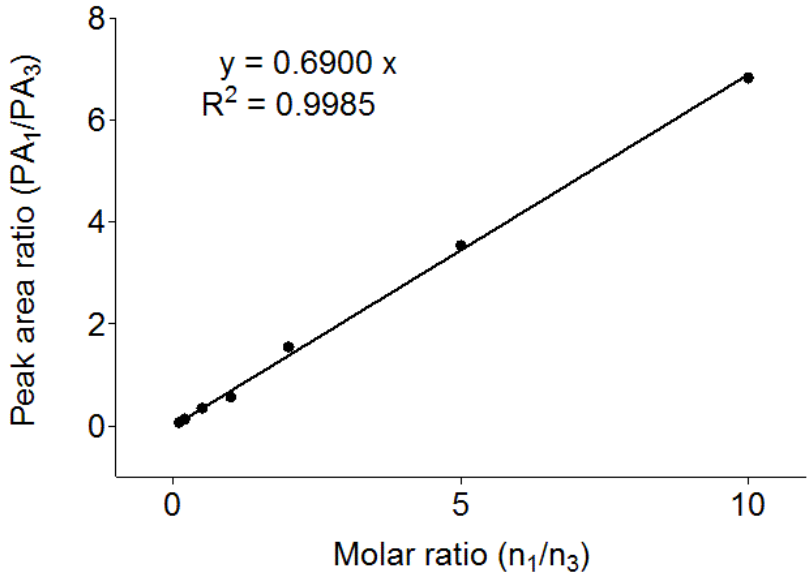

Fig. S17 Determination of the correction factor. The HPLC ratio of peak area (PA_1/PA_3) were determined with the standard molar ratio (n_1/n_3) of 1/10, 1/5, 1/2, 1, 2, 5 and 10. The correction factor (f = 0.69) was estimated from the fitting curve (R^2 = 0.9985).

(a)

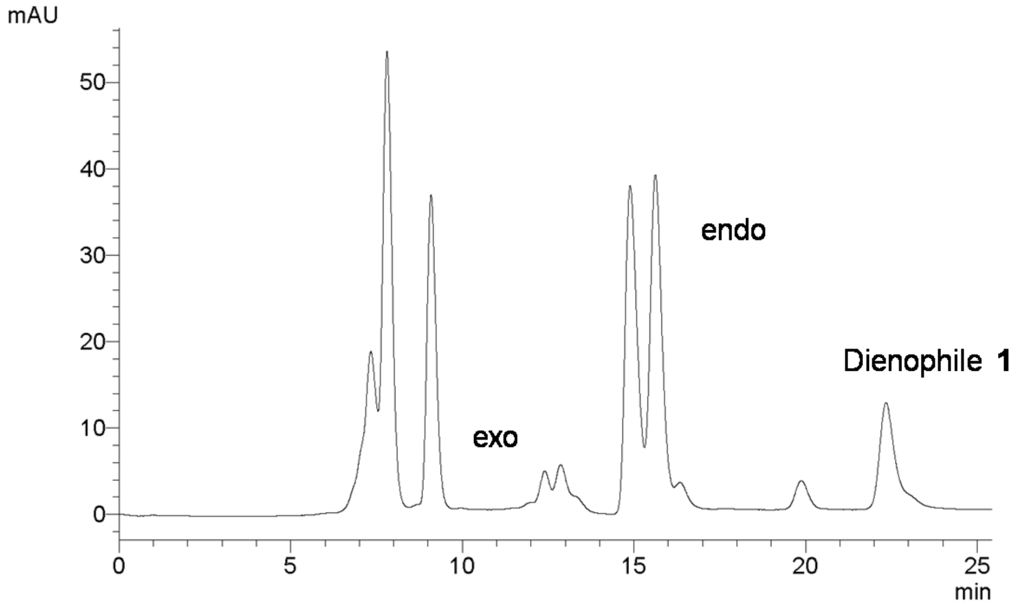

(b)

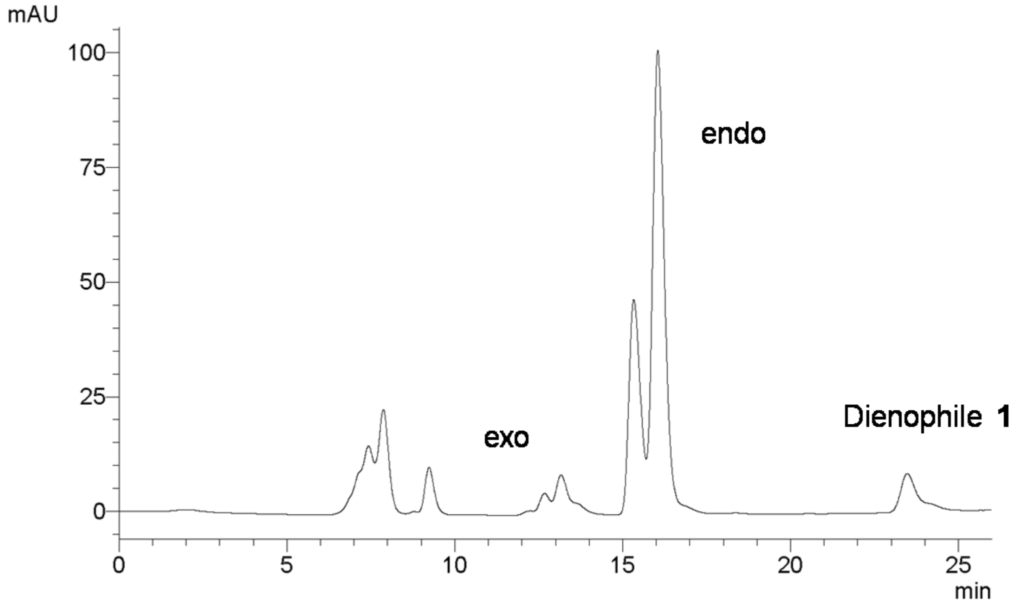

Fig. S18 Typical HPLC traces of the crude products from Cu^{2+} catalysed Diels-Alder reaction. (a): Cu^{2+} without duplex **8a**/**8b**, negative control, entry 2 in Table 2. (b): Cu^{2+} with duplex **8a**/**8b**, entry 1 in Table 2. Condition: heptane/2-propanol 99:1, 0.5 mL/min, column temperature 40 °C.

NMR and ESI data

^{1}H-NMR spectra were recorded in deuterated solvents on Bruker ARX 300 and Bruker AMX 600 spectrometers and calibrated to the residual solvent peak. Chemical shifts (δ, ppm) are quoted relative to the residual solvent peak as internal standard and coupling constants (*J*) are corrected and quoted to the nearest 1 Hz (or 0.1 Hz). Multiplicities are abbreviated as follows: s = singlet, d = doublet, t = triplet, m = multiplet.

3-phenyl-1-(2-pyridyl)prop-2-en-1-one (1)

Spectroscopic data in agreement with those reported in S. Otto, F. Bertoncin, J. B. F. N. Engberts, *J. Am. Chem. Soc.* **1996**, *118*, 7702-7707.

^{1}H NMR (300 MHz, $CDCl_3$): 8.75 (m, 1H), 8.32 (d, *J* = 15 Hz, 1H), 8.20 (m, 1H), 8.00 (d, *J* = 15 Hz, 1H), 7.89 (m, 1H), 7.74 (m, 2H), 7.49 (m, 1H), 7.42 (m, 3H).
^{13}**C NMR** (75 MHz, $CDCl_3$): 189.3, 154.1, 148.7, 144.8, 137.1, 135.1, 130.6, 128.8 (4C), 126.9, 122.9, 120.9.
HRMS (ESI+): calculated for $C_{14}H_{12}NO^+$ $[M+H^+]^+$: 210.0913; found: 210.0912.

(3-phenylbicyclo[2.2.1]hept-5-en-2-yl)(pyridin-2-yl)methanone (3)

Spectroscopic data in agreement with those reported in S. Otto, F. Bertoncin, J. B. F. N. Engberts, *J. Am. Chem. Soc.* **1996**, *118*, 7702-7707.

^{1}H NMR (600 MHz, $CDCl_3$): 8.69 (m, 1H), 8.02 (d, *J* = 7.9 Hz, 1H), 7.84 (t, *J* = 7.9 Hz, 1H), 7.47 (m, 1H), 7.30 (m, 4H), 7.16 (t, *J* = 7.2 Hz, 1H), 6.50 (s, 1H), 5.83 (m, 1H), 4.55 (m, 1H), 3.55 (s, 1H), 3.45 (d, *J* = 4.8 Hz, 1H), 3.09 (s, 1H), 2.08 (d, *J* = 8.4 Hz, 1H), 1.60 (d, *J* = 8.4 Hz, 1H).
^{13}C NMR (150 MHz, $CDCl_3$): 200.8, 153.3, 148.6, 144.5, 139.5, 137.1, 132.8, 128.4 (2C), 127.6 (2C), 126.9, 125.8, 122.3, 54.3, 49.4, 48.7, 48.2, 45.6.
HRMS (ESI+): calculated for $C_{19}H_{18}NO^+$ $[M+H^+]^+$: 276.1388; found: 276.1382.

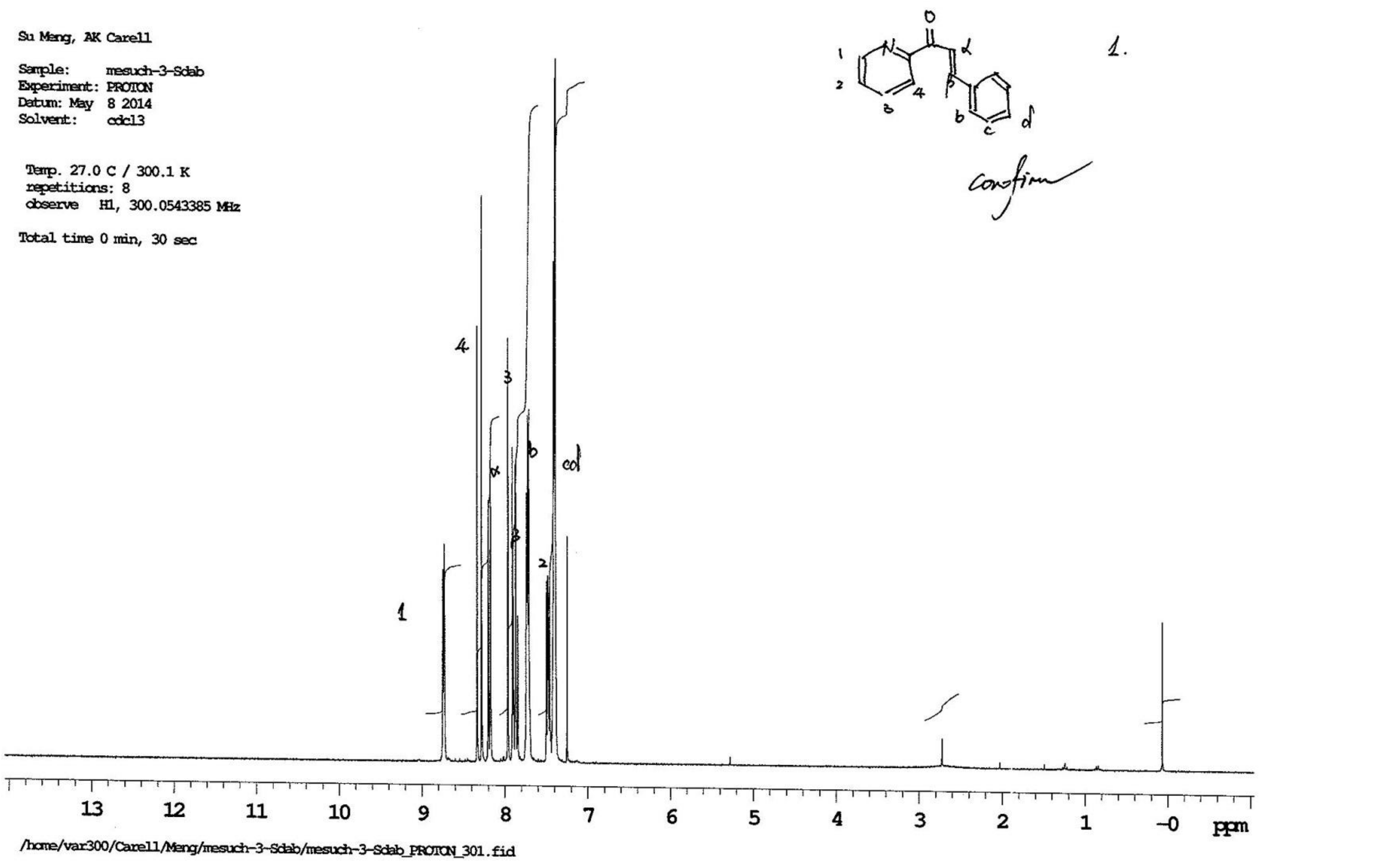
Su Meng, AK Carell
Sample: mesuch-3-Sdab
Experiment: PROTON
Datum: May 8 2014
Solvent: cdcl3
Temp. 27.0 C / 300.1 K
repetitions: 8
observe H1, 300.0543385 MHz
Total time 0 min, 30 sec
1.
confirm
13 12 11 10 9 8 7 6 5 4 3 2 1 -0 ppm
/home/var300/Carell/Meng/mesuch-3-Sdab/mesuch-3-Sdab_PROTON_301.fid

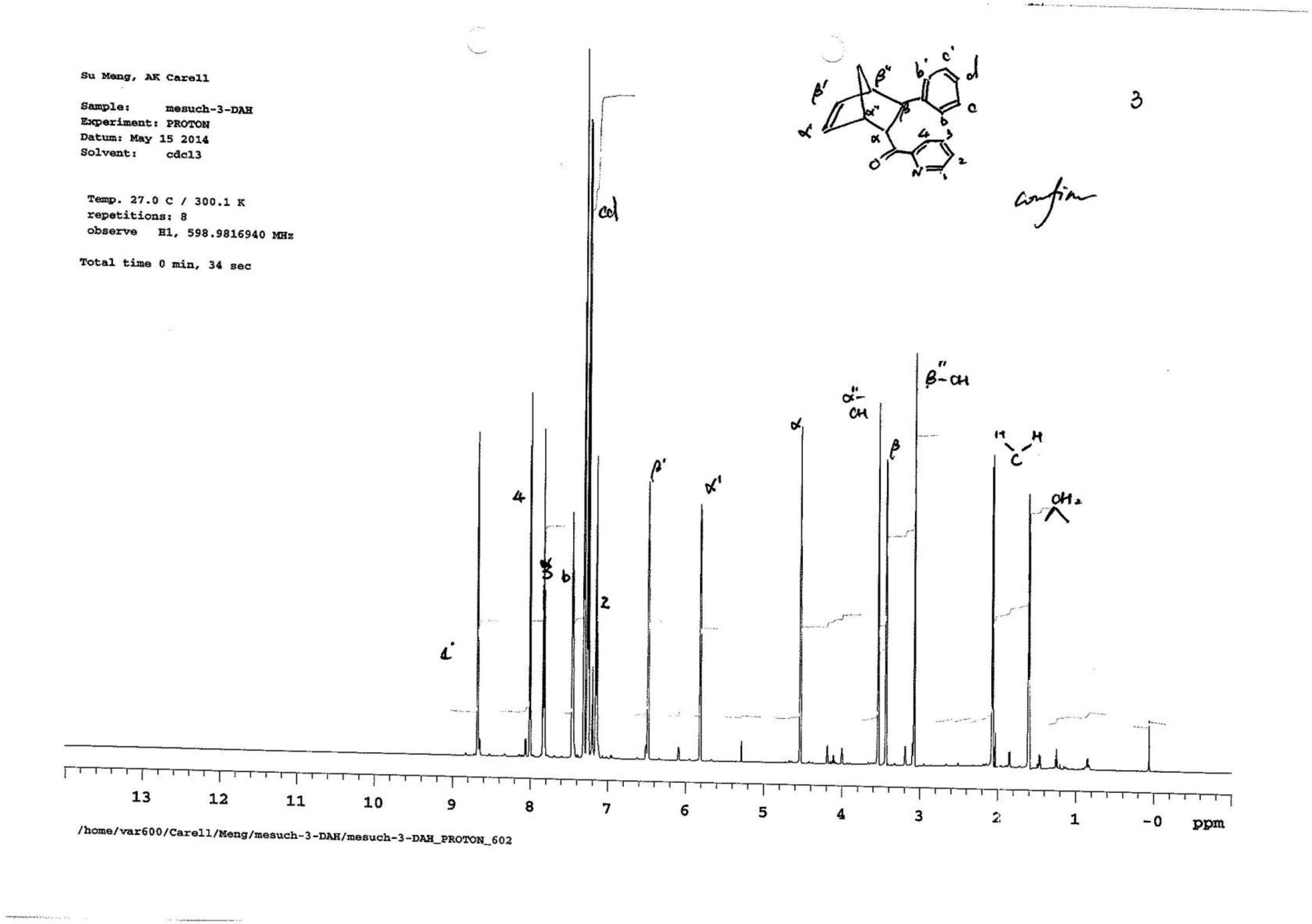
Su Meng, AK Carell
Sample: mesuch-3-DAH
Experiment: PROTON
Datum: May 15 2014
Solvent: cdcl3
Temp. 27.0 C / 300.1 K
repetitions: 8
observe H1, 598.9816940 MHz
Total time 0 min, 34 sec
3
confirm
cdl
13 12 11 10 9 8 7 6 5 4 3 2 1 -0 ppm
/home/var600/Carell/Meng/mesuch-3-DAH/mesuch-3-DAH_PROTON_602
